# Challenges and opportunities for medical referrals at a mobile community health clinic serving sexual and gender minorities in rural South Carolina: a qualitative approach

**DOI:** 10.1186/s12913-023-09141-z

**Published:** 2023-02-17

**Authors:** Layla Joudeh, Smith F. Heavner, Ethan Johnstone, Shantara K. Propst, Orlando O. Harris

**Affiliations:** 1 School of Medicine, University of California, San Francisco, USA; 2 Department of Public Health Sciences at, Clemson University, Clemson, SC USA; 3 Department of Biomedical Sciences, University of South Carolina School of Medicine Greenville, Greenville, SC USA; 4 Harvard Medical School, Boston, MA USA; 5 CDRC, Critical Path Institute, Tucson, AZ USA; 6 Pride Link Mobile Community Health Clinic, Greenville, SC USA; 7 Edward Via College of Osteopathic Medicine- Carolinas, Spartanburg, SC USA; 8 Department of Community Health Systems, School of Nursing, University of California, San Francisco, USA; 9 Department of Medicine, Center for AIDS Prevention Studies, University of California, San Francisco, USA

**Keywords:** Medical referrals, Sexual and gender minority, Mobile clinic, Southern United States

## Abstract

**Background:**

Sexual and gender minorities (SGM) in the Southern United States face challenges in accessing sexual and gender affirming health care. Alternative care models, like inclusive mobile clinics, help mitigate barriers to care for SGM. There is limited data in the literature on the experience of medical referral processes for SGM individuals accessing services from mobile health clinics.

**Aims and objectives:**

The purpose of this study is to describe the medical referral experiences of SGM clients and their providers at a mobile health clinic in the Southern United States.

**Methods:**

We recruited English-speaking individuals who provided care or received care from the mobile health clinic in South Carolina between June 2019 and August 2020. Participants completed a brief demographic survey and a virtual in-depth, semi-structured individual interview. Data analysis was conducted using an iterative process to generate codes, categories, and themes. Data collection and analysis were terminated once thematic saturation was achieved.

**Results:**

The findings from this study indicated that the mobile health clinic had an inconsistent referral process that was largely dependent on providers’ knowledge. Furthermore, clients and providers expressed individual barriers to the referral process, such as financial barriers, and opportunities to improve the referral process, such as an opt-in follow-up from the mobile clinic and increased mobile clinic resources.

**Conclusion:**

The findings in this study underscore the importance of having mobile clinics create a structured referral process that all medical providers are familiar with, and the value of hiring patient navigators that can support and refer clients to care that goes beyond the mobile health clinic setting.

## Background

Sexual and gender minorities (SGM) encounter many social and structural barriers in accessing quality affirming healthcare in the Southern United States (U.S.) [1]. Sexual minority is defined as a person who does not exclusively identify as heterosexual, which includes those who identify as lesbian, gay, bisexual, questioning, or intersex. Gender minority is someone whose gender identity does not align with the sex that they were assigned at birth, which include those identifying as transgender or nonbinary [2]. The barriers persons from these marginalized groups experience include higher rates of stigma and discrimination, increased poverty, homelessness, underemployment resulting in lower wages, violence, and food insecurity—all of which are significantly associated with disparities in healthcare access and outcomes [3]. Additionally, SGM in the Southern U.S. are less likely to be insured compared to SGM individuals in other parts of the country [1, 4, 5], a direct result of most states in the South not expanding insurance coverage through the Patient Protection and Affordable Care Act of 2009. To address the deficit in accessing quality healthcare, many SGM who live in the South find alternative ways to access health care, including mobile health clinics as opposed to brick and mortar clinics like hospitals or private practice offices [6–8].

While mobile clinics have several advantages in providing care to neglected communities, they cannot guarantee continuity of care and are dependent on relationships with other hospitals and specialty care centers to ensure continued care for their clients [9, 10]. Mobile health clinics are defined as easily transportable clinic units that offer services at different locations in a community [10]. The most common services offered in mobile health clinics in the U.S. are primary and preventative care (e.g., sexually transmitted infection (STI) testing, cervical cancer screening, breast cancer screening, blood pressure screening) [11]. They have been shown to reduce emergency department visits, reduce hospitalization costs, and increase symptom-free days for pediatric asthma patients—all of which reduce healthcare expenditures [10]. However, follow-up care has proved challenging. In one study involving a mobile clinic with a focus on specialized wound care, the authors reported on the challenges of retaining, referring, or following up with their clients after their mobile clinic encounters [12]. Referral to care is also imperative for mobile clinics screening for human immunodeficiency virus (HIV) [13]. Mobile clinic staff are encouraged to make referrals for clients to link them to specialty providers (i.e., infectious disease clinicians) for continued HIV care—which often results in many clients not being properly linked to and retained in vital HIV treatment programs [13]. Effective referral to care is especially important in the southern United States where there is the highest concentration of HIV infection [14, 15].

There is limited data in the literature on the experiences of sexual and gender minority patients accessing care from mobile health clinics as well as their experiences with navigating the medical referral process in order to ensure continuity of care beyond the limitations of the services offered within mobile health clinics. Moreover, little is known about SGM individuals’ experiences with the medical referral process of mobile community clinics as well as the experiences of the providers making those referrals. The experiences of the clients are intertwined with the providers who are tasked with making the medical referrals. Thus, the purpose of this qualitative descriptive study was to describe the medical referral experiences of SGM clients and their providers at a mobile health clinic in South Carolina and to identify strategies to improve the medical referral process.

## Methods

We employed a qualitative descriptive approach to evaluate the medical referral process at a local MCHC in rural South Carolina in 2020. We conducted individual, semi-structured, open-ended interviews with MCHC clients and the MCHC healthcare providers to understand their experiences with the medical referral process. Due to the COVID-19 pandemic, all study procedures were conducted virtually using a secure video platform. The study received expedited oversight approval (#20–30440) from the University of California, San Francisco Institutional Review Board (IRB).

### Positionality and reflexivity

The authors of the present study represent different demographic backgrounds and brought their unique positionalities to the research project. To limit biases and ensure rigor, the research team was multidisciplinary, held different identities, and engaged in a systematic approach to data collection and analysis. The authors also reflected on potential biases throughout the study process (i.e., data collection and interpretation of the results) and adjusted procedures to limit them. For example, the lead author was the primary person interfacing with the participants as she had no other connection with them compared to the MCHC collaborators, and during data analysis, the MCHC collaborators were primarily involved in expert validation via peer debriefing of the codes, categories, and themes [16, 17]. The lead author is a medical student and woman of color, who was raised in South Carolina. The second author is an evaluation scientist and registered nurse. The middle authors either volunteered or worked within the MCHC and are referred to as MCHC collaborators in the manuscript. The senior author is a health disparities researcher whose research focuses on marginalized and minorized populations in the United States and the Caribbean.

### Setting

The MCHC is a mobile community clinic that provides free healthcare to SGM individuals in rural South Carolina. The MCHC is a 501c3 non-profit organization that provides free health services with funding from grants, local partnerships, and individual donors. The MCHC prioritizes gender inclusive and affirming healthcare services, such as mental and physical health screenings, testing for STIs, HIV, and hormone therapy resources. They also provide assistance with obtaining state benefits, legal name changes, and spaces for socialization. While the MCHC specializes in care for SGM individuals, sexual and gender identity does not determine whether someone can access services. The organization serves between five and 50 clients per month by traveling to different towns. Prior to the COVID-19 pandemic, they offered monthly in-person services in alternating towns. However, in the first several months of the pandemic, they offered monthly in-person or virtual services depending on public health protocols. The medical providers at the MCHC are all volunteers with the exception of the specialized HIV providers. The HIV providers are employed by an organization that provides full spectrum HIV services at health fairs and mobile clinics. On average, there are six to 12 medical volunteers present at every MCHC session. Volunteer medical staff consist of nurses, physicians, mental health providers, phlebotomists, social workers, HIV counselors, medical and osteopathic medicine students, and non-medically affiliated individuals.

### Participants

We recruited clients and health care providers to participate in the study between June and August 2020 using purposive sampling techniques. Healthcare providers were eligible to participate if they were at least 18 years of age, had provided healthcare services in an in-person setting at the MCHC at least once since June 2019, were not part of the research team, and could complete study procedures in English. MCHC clients were eligible to participate if they were 18 years of age, able to complete study procedures in English, and had used any of the services offered at the MCHC at least once since June 2019. Sexual orientation and gender identity were not included as part of the inclusion or exclusion criteria. Our priority was to recruit individuals who received services or provided services at the MCHC—which centers SGM individuals but does not deny care based on sexual and gender identity. Given the risk for stigma and violence towards SGM individuals, we did not want to exclude individuals who did not feel comfortable identifying as SGM but still valued and utilized the services offered by the MCHC.

### Procedures

Our procedure for research engagement, data collection, and analysis employed a collaborative approach with members of the MCHC community. While we did not use a comprehensive community-based participatory research (CBPR) approach, we utilized key components of it to guide our study design and procedures [18, 19]. The MCHC collaborators played key roles in designing the study, participant recruitment and engagement activities, data analysis, and manuscript preparation.

#### Recruitment

The lead author conducted purposeful sampling recruitment activities using several methods to ensure outreach to as many eligible individuals as possible. First, we conducted convenience sample recruitment using email outreach to a client and provider database and social media posts to the MCHC social media pages. Second, we purposefully sampled gender, racial, and ethnic minorities via email in order to ensure a diverse sample. We finally conducted snowball sampling via peer referrals by asking previously interviewed participants to share the study contact information with others in their network who also access or provide services at the MCHC [20, 21]. Once a potential participant interfaced with recruitment material, they were asked to contact the lead researcher via telephone if they were interested in the study. Then the potential participant was screened for eligibility using the inclusion criteria. Those who met the eligibility requirements were verbally consented and invited to complete the study’s demographic survey and individual interview over the phone or video communication service. The lead author conducted all the interviews. Participants received a $35.00 electronic gift card honorarium for their time. To maintain confidentiality and limit the potential for bias, the MCHC collaborators were not involved in the screening and consenting of potential participants.

#### Survey and interview guide

A brief survey was administered to clients and providers prior to each individual interview to obtain demographic information. The demographic survey data included age, race, gender, sexual orientation and highest level of education. Clients were asked additional questions about income, insurance type, and their access to a primary care provider (see Table 1). Two in-depth, semi-structured interview guides were created ahead of time to guide the individual client and provider interviews. The main topics discussed in the interview guide were client and provider experiences within the MCHC, medical referrals at the MCHC, and existing or potential supports for clients and providers. See Table 2 for a description of the content areas as well as sample questions for both the client and provider interviews.

**Table 1:** Demographic characteristics of study participants

*n* (%) Unless Otherwise Noted	Clients (*N* = 12)	Providers (*N* = 8)
Mean age in years (± *SD*)	27.3 ± 4.44	35.5 ± 11.3
Race		
Black	5 (42)	1 (12)
Hispanic	1 (8)	0 (0)
White	6 (50)	7 (88)
Mean annual income		
$20,000 or less	1 (8)	
$20,001 to $40,000	4 (33)	
$40,001 to $60,000	5 (42)	
$60,001 +	2 (17)	
Type of insurance		
Private non-employer based	4 (44)	
Employer-Based	6 (50)	
Medicaid/Medicare	1 (8)	
No insurance	1 (8)	
Have a primary care provider	7 (58)	
Sexual orientation		
Queer	3 (25)	0 (0)
Bisexual	1 (8)	0 (0)
Pansexual	2 (17)	0 (0)
Lesbian	2 (17)	1 (12)
Gay	3 (25)	1 (12)
Heterosexual	1 (8)	6 (75)
Gender identity		
Non-binary	2 (17)	0 (0)
Transgender female	1 (8)	0 (0)
Transgender male	3 (25)	0 (0)
Cisgender female	3 (25)	6 (75)
Cisgender male	3 (25)	2 (25)
Highest level of education		
Some college	4 (33)	0 (0)
Two-year associate degree	1 (8)	0 (0)
Four-year bachelor’s degree	6 (50)	2 (25)
Graduate or professional degree	1 (8)	6 (75)

**Table 2:** Sample questions for interview content areas

Interview Content Area	Sample Question for Client	Sample Question for Provider
Experiences with the MCHC	Why did you seek services at the MCHC?	What was your role with the MCHC?
	Could you describe what your general understandings are of the purpose of the MCHC?	What is your understanding of the purpose of the MCHC?
	What was challenging about accessing services at the MCHC?	What did you like or dislike about your experiences with the MCHC?
Medical referrals	What are some obstacles for you when you are trying to follow through with a medical referral?	In what ways do you think you have used medical referrals at the clinic?
	What were things you want to make sure providers you referred to were able to do?	What have clients communicated to you about the referral system?
	Were there any other community resources available for you when you were trying to find care or follow through with a referral?	Have they identified any barriers specifically to you? What were some of those barriers?
Supports in accessing or providing care	What would be helpful for health care providers and offices to do to make people feel comfortable going to see a provider?	How do you envision the MCHC having a role in following up with clients after a referral?
	Are there resources in your area that help SGM individuals find resources?	What did you think about the onsite resources? What are the strengths of these resources? What are the limitations of these resources?
	Why is it challenging to find SGM-specific support resources?	Based on your experiences with the MCHC, what would be some of the life obstacles for clients to follow through with referrals?
	How do you go about finding an affirming provider?	
	What do you think would be important for creating a list of affirming providers?	

#### Data collection and analysis

Survey data was collected and stored using REDCap software. Survey questions were read aloud to the participants and the lead author completed the REDCap survey based on participants’ verbal responses. Interviews were audio and video recorded, deidentified with a generic numeric code, and transcribed verbatim by a professional transcriptionist who is trained in human subjects protection and confidentiality. The length of the interviews ranged from 30 to 90 min. ATLAS.ti version 9.0.3, a qualitative data software, was used to store and manage the interview data. Both the lead and senior author, a qualitative expert, conducted the qualitative data analysis. Code development and extraction involved an iterative process that included consultations with qualitative experts, such as the senior author and those familiar with the research topic area to ensure consensus [22]. A codebook was created and shared with the senior author to ensure the codes captured the essence of participants’ narratives. Both the lead and senior author further coded large portions of text that represented key ideas. Initial codes were compared for similarities and terminated based on repetitiveness. Code refinement continued by clustering similar codes into categories [23]. Categories were then expressed as themes [22–24]. To ensure expert validation, all the authors discussed the themes, the interpretation of the exemplar quotes, and clarified the themes based on the MCHC collaborators’ expertise and familiarity with the population [17, 22]. Peer debriefing with the senior author and MCHC collaborators contributed significantly to the trustworthiness and validity of the study findings [25]. Both data collection and analysis continued until saturation was achieved. Saturation was achieved after no new information was generated from conducting interviews, coding narratives, and categorizing codes [26].

## Results

### Participant characteristics

Participants’ (*N* = 20) demographic characteristics are displayed in Table 1. The mean age of participants was 27 and ranged from 24 to 35 years of age. The mean age for providers was 35 and ranged from 25 to 60 years of age. Providers included two master’s level licensed counselors, a licensed clinical social worker, a certified HIV counselor who also served as a phlebotomist, two licensed medical doctors, and two osteopathic medicine students. All participants self-described their race, sexual orientation, and gender identity with three open-ended survey questions. Of the clients, five identified as Black (42%), six identified as White (50%), and one identified as Hispanic (8%). Of providers, one participant identified as Black and the other seven participants identified as White. 92% (*n* = 11) of client participants had a non-heterosexual sexual orientation, 50% of clients identified as cisgender (*n* = 6), and 50% were transgender or nonbinary (*n* = 6). Two of the providers (25%) had a non-heterosexual sexual identity, six providers identified as ciswomen (75%), and two identified as cismen (25%).

### Central themes

The data presented highlight four central themes and eight additional subthemes that emerged after we completed the analysis of the data. In the subsequent results section, we outlined the four primary themes and the eight subthemes along with several exemplar quotes that arose from MCHC clients and providers around their experiences with the MCHC medical referral process. Table 3 provides details about the themes and subthemes as well as additional exemplar quotes. To protect participants identity, we use pseudonyms with the corresponding quotes below.

**Table 3:** Thematic categories with themes, subthemes, and exemplar quotes

Theme	Subtheme	Exemplar quote
Inconsistency in referral process	Varying referral types	“[There are] three or four … practitioners in [city] that I trust and then [I made] sure it was okay to refer to myself … It was really [figuring out how I will refer] on-site and just going ahead and coming up with resources that I already knew of. Or resources that other people at [the MCHC] knew of and … just asking and checking, ‘hey, are these appropriate?’”
	Lack of infrastructure	“I got there an hour earlier so that I could be prepped and set up. But there wasn't a formal orientation ahead of time or anything like that.”
Provider referral resource knowledge	Professional network	“[The mental health screener] recommended a [local therapist]. I reached out to [them], and we set our initial consultation which was probably two or four weeks after I went to the [MCHC] … It's been a little over year, [they’re] still my therapist.”
	Sponsoring organization	“The soft referral and the handoff is basically within our own organization. And with all of our forms and everything, and even with referrals, we get individuals to sign an authorization so that we can make that referral to another provider.‬‬‬”
Individual client barriers in the referral process	Resource limitations	“For some individuals, especially those that work, having to get off work to go to appointments [is an obstacle] because not everybody has the type of job where they have sick leave or [paid time off].”
	Anxiety around healthcare systems	“A major issue … [is] if it's not an obviously affirming [provider], …because like if I send a referral to [a clinic,] they're not necessarily affirming or non-affirming. They're just the place to go if you need to see a psychiatrist and a counselor…so, depending on how sensitive [a client is] to people not being super affirming and understanding, that could be a deterrent.”
Opportunities for improving the client referral process	Follow-up with clients	“It’s the gaps that people can fall through. In [South Carolina] being a queer person seeking medical services [means] there are a lot of challenges. And so, anything to close those gaps and reach out and make sure that people are taken care of shows a great deal of respect and care for the community.”
	Resource availability	“[Getting] local doctors when [the MCHC] does stuff would probably help because then [I am] able to [assess if], I really like [the] person. Let me see if I can schedule something with them or … they're able to link you up with somebody near because they're in that community.”

### Inconsistency in the referral process

The theme around the inconsistency of the referral process captures the reality of providers and clients having mixed experiences with receiving and providing medical referrals. A pervasive narrative that outlines those inconsistencies were also reflected in two of the subthemes, *varying referral types* and *lack of infrastructure* to support the referral process.

#### Varying referral types

The subtheme *varying referral types* highlights a number of ways referrals are made within the mobile health clinic. Clients and providers described three main types of referrals. Clients can receive an on-site appointment made with a MCHC provider with a practice in the community or volunteer organization, a written resource suggesting where to seek follow-up care, or a verbal suggestion from providers as to where a client can seek follow-up care. While these varying referral types offered inconsistency with the referral process, some clients described the helpfulness of a follow-up appointment being scheduled for them while they were at the MCHC with the same provider with whom they interacted while at the MCHC. For example, one client described their satisfaction with being able to set up a follow-up appointment with the same mental health therapist they saw at the MCHC. They stated:

It was comforting. And it was [helpful] to be able to [have access to a] resource that I've been looking for . . . And I think the best thing of all was the ease . . . I was able to do the screening and then immediately set-up a follow-up appointment. (Rodrigo)

Another client was provided a referral to a mental health therapist at the mobile clinic, but they were asked to make their own appointment, which was another example of the varying referral process. Charlie described their experience in the narrative below:

[The counselor I saw at the MCHC] said, ‘here’s my office, here’s the phone number, why don’t you give us a call and let’s set something up.’ She had all the information herself and knew what the costs were … I think that that was really one of the best things that has come from the mobile community center. (Charlie)

Other clients were given referrals to providers that were new to them and asked to schedule their own follow-up appointments. Some clients expressed appreciation for the verbal referral and were successful in following up (i.e. setting up an appointment where they were referred) on their own. However, others were not able to connect to care with a verbal referral as no appointment was set up for them. Those clients who did not receive either a verbal or written referral, but rather collected a community resource sheet located within the mobile clinic mentioned misplacing the resource sheet or not being able to located it when they were in need of services such as mental health care.

Parallel to clients’ experiences, providers discussed their experiences with providing clients verbal referrals. They employed this referral type in order to make recommendations to clients for continued care with community specialist. One provider described a treatment that he offered to client who then expressed interest with continuing that type of treatment. Owen, the provider at MCHC expressed:

I was doing OMT [osteopathic manipulative treatment] on a patient and they really enjoyed it and they [said], "this is something I could use on a regular basis." So, I gave them [the] information [for an OMT provider]. (Owen)

##### Lack of infrastructure

The next subtheme that describes the inconsistency with the referral process was centered around the *lack of referral infrastructure*. The narratives from clients and providers highlighted the lack of referral infrastructure at the MCHC, which limited some providers’ abilities to give comprehensive referrals. Several of the providers reported frustration in the limited resources they had to assist clients continue their care outside of the limited MCHC setting. One provider expressed frustration with not having the resources to follow up with and support their clients—"I couldn’t [make an actual referral]... It was kind of like me just saying, 'hey, I think you should do this, but I can’t help you do that.'” Some of the frustration that the provider expressed included not having the ability to use an electronic health record or an actual referral system to make a formal medical referral. Providers also expressed that there was no established MCHC procedure or policy in place to allow providers to follow up with clients, even after a referral (verbal or written) was made. Volunteer providers also noted that there was no training from the MCHC staff on how to provide referrals to the clients that were being seen at the MCHC.

### Provider referral resource knowledge

The next theme that emerged from the data centered around provider referral resource knowledge. This theme captured the nature of the referral process and how it was contingent on the providers’ knowledge of community medical resources that were available at their disposal. The narratives around provider referral resource knowledge was also reflected across two subthemes, knowledge of other providers in their professional network and the resources available within their sponsoring organization.

#### Professional networks

The subtheme around provider professional network highlights how providers relied on their own professional networks when deciding to whom they would refer clients seen at the MCHC. Providers’ narratives demonstrated the importance of knowing local community providers, more specifically SGM-affirming healthcare providers. For example, one of the providers discussed how their time practicing in the community helped them make referrals – “I did my [training] and I practiced [in this area] for years. That’s given me the chance to build a reputation here and also know the players in the game as far as the community goes.” While another provider stated that they did not need training on community resources when making referrals due to their prior experience, “I think I just already knew how to [give a referral] because of my prior experience. So that was easy to do.”

#### Sponsoring organizations

The other subtheme illustrating provider referral resource knowledge was their employment or affiliation with their *sponsoring organization*. The provider’s affiliated or sponsoring organization affected the referral process in terms of capacity of the institution or their ability to accept new clients. For example, the primary mental health provider volunteers at the MCHC expressed that they were affiliated with an organization who had the capacity to take on new clients. Often, they were able to immediately schedule a client referral appointment. One of those mental health providers shared their experience around their process for referring clients to their sponsoring organization:

Once we do a screening and we see that … [the client] would probably benefit from additional services, we have our business cards there … So, we have plenty of therapists that are [at the MCHC] that could likely take on new clients. So, we connect them and try to get them on a schedule. (Ellis)

Another community healthcare organization that partners with the MCHC had formalized internal procedures for referrals because their organization was designed to provide comprehensive care and follow-up in settings like the MCHC. The provider described how their organization has multiple levels of follow-up for clients who receive a positive HIV test result at the MCHC.

### Individual client barriers in the referral process

The third theme, *individual client barriers in the referral process*, which emerged from both client and provider narratives highlighted participants difficulty with the referral process. Their experiences also highlighted client-level barriers in navigating referrals within the MCHC and the community. The narrative around the individual client barriers in the referral process also illuminated two additional subthemes, resource limitations and the anxiety they experience around accessing care from unfamiliar healthcare systems.

#### Resource limitations

*Resource limitations* was represented in providers’ and clients’ narratives that described logistical barriers to scheduling and following up with referral appointments. The main barriers were the lack of financial resources and not having enough time to make medical appointments. Coincidentally, the provider experiences with MCHC clients also mirrored these two challenges. One client who expressed financial and scheduling challenges in following up with a referral appointment that was made for them stated “very specifically, it’s financial. It’s always been very tough [because]... I don’t have anybody to help with bills... and basically just finding time to [go to the appointment is challenging].” In addition to financial burdens, some providers expressed that many of their clients reported not being able to miss a day from work or take time off work for medical appointments (in contrast to the weekend hours of the MCHC) due to the financial impact.

#### Anxiety around healthcare system

For some clients, there was anxiety around accessing healthcare from an unfamiliar medical environment. A major contributor to this anxiety stems from clients’ concerns around how they will be treated as a sexual or gender minority. Due to prior experiences with stigma and discrimination, some SGM were concerned around whether or not the new facility would acknowledge them by their correct name or pronouns. These concerns also had a psychological impact on the clients who are attempting to access external healthcare. For example, one client described multiple challenges when entering a clinical space as someone who identifies as SGM, including how they have been treated in the medical space:

You’re trusting the referral process . . . [that] the computer doesn’t glitch…they spell your name right …or entered the right pronoun . . . I think after that there’s just like the working up the mental energy to enter a new medical environment. (Lilly)

Another client found it challenging to independently navigate mental health care for the first time, since their family was not supportive of care related to their SGM identity– “It was a little nerve-wracking... I’ve been doing therapy … but [this] was honestly the first time that I had [to make] my own appointment... So, I was very nervous trying to set things up myself.” Although this client had the added benefit of having previously interfaced with the mental health provider to which they were subsequently referred to, calling them to follow through for continued care was still a challenging experience for them.

### Opportunities for improving the client referral process

The final theme that emerged from the data offered opportunities for improving the client referral process at the MCHC. The narratives that materialized from this theme also highlighted two additional subthemes, a process for following up with clients and the resource availability. Through analysis of the collective narratives offered from clients and providers, several possibilities for improving the referral process was offered. For example, both clients and provides made two salient recommendations: (1) for staff to follow up with clients to learn whether a referral was helpful and (2) provide any additional support that the client may have needed after they visited the mobile health clinic. For example, one of the clients described how a follow-up call with an MCHC staff member after a referral would be helpful for understanding how the MCHC could improve referrals:

It would be good to see if people did follow through with their appointment or if they weren’t able to have the continuation of care. If they weren’t able to, would the mobile clinic be able to help them? It would be important to see what the success rates were for medical referrals made. And if the client didn’t go, why? (Cade)

The same client explained that it is important for the SGM community to have additional support after a referral because queer individuals often face unique challenges when seeking medical services.

While some participants discussed that a follow-up conversation after a MCHC visit would be helpful, others were concerned about the confidentiality of a follow-up call and whose role it would be to follow up with clients. One client discussed the tension that could exist if the mobile clinic staff contacted a medical provider without them knowing or anticipating that call:

It should be opt-in because you don’t want to scare people away. You don’t want people to feel obligated that if they come to the clinic … the clinic would do something with the information afterwards. (Charlie)

Providers and clients recommended having a list of SGM affirming resources available to help navigate the referral process. They suggested the resource include a list of sexual and gender minority affirming providers. One of the providers in our study explained that because the mobile clinic provides care in different towns, a list of well-vetted affirming providers can ensure that providers give referrals beyond their own professional network. Clients expressed how having a referral to a MCHC provider would lead to more effective health care provision. One improvement would be referring clients to the providers they saw at the MCHC. One client stated:

[It] was a little bit difficult to follow up with a non-MCHC provider. I explained to [the non-MCHC provider] that I had gone to a different clinic . . . and they said, ‘come in three months’. . . and that was a little frustrating because they told me at the [MCHC] that I needed to be seen right away. If the people who were doing the medical screenings were the same people I had to follow up with, then [my blood pressure screening] might have been taken more seriously. (Charlie)

The findings presented thus far reflected clients and providers experiences with the referral process. These experiences stemmed from inconsistency with the referral process, provider referral resource knowledge, the barriers clients experience when accessing referral services, and opportunities for improving the referral process.

## Discussion

Our study aimed to understand the medical referral process and experiences for health care providers and SGM individuals at a MCHC in the Southern U.S. The four primary themes that emerged from client and provider narratives were inconsistency in the referral process, provider referral resource knowledge, individual client barriers in the referral process, and opportunities for improving the client referral process. Our findings demonstrated that referrals made from mobile community clinics to specialized or primary care providers were often inconsistent—with clients receiving referrals in multiple ways and providers giving referrals in multiple ways. This inconsistency was often rooted in the lack of referral infrastructure at the MCHC. Provider’s professional networks and the organization’s sponsoring the providers also affected the referral experiences of providers and clients. Finally, there were individual client barriers that affected how clients engaged with a referral once it was given—including financial burdens and anxiety around engaging with a new medical space.

Notably, our study aimed to understand the medical referral process in an SGM specific setting, and only one theme, individual client barriers, directly reflected challenges that clients face directly due to their SGM identifies. However, SGM individuals are more likely to seek alternative types of care, like the SGM-centered MCHC, due to stigma and discrimination [6–8]. Thus, while some of our findings might apply to non-SGM centered alternative modes of health care provision, SGM individuals are disproportionately affected by the gaps in care at mobile clinics.

Clients who accessed services from the mobile clinic spoke specifically to a referral process that was dependent on what an individual provider could offer in the moment. These findings align with previous literature demonstrating that a key limitation of mobile clinics is their inability to offer continuous care [10]. An analysis of mobile health clinics in a suburban county in California demonstrated that mobile clinics experience challenges in facilitating clients’ access to specialty care such as gynecology, endocrinology, and gastroenterology [27]. The literature also demonstrates that mobile clinics have challenges promptly linking high-risk sexually active individual who test positive for HIV in mobile community outreach vans to care [13]. In our sample, clients preferred receiving specialized care referrals at the mobile clinic as opposed to having to find specialty care on their own. This preference for an on-site referral mirrors findings from the literature showing clients want centralization of their care (i.e. a one-stop shop) [28, 29].

One reason the MCHC demonstrated an inconsistent referral process is because it is not integrated into an existing health care system, as seen in other models of mobile health clinics [10, 27]. A survey of the literature on mobile clinic models revealed that many mobile clinics tried to overcome this challenge by contracting with an established health care system thus more easily facilitating continuation of care for clients [12, 30]. This is also a model endorsed in HIV care provision [13, 31, 32]. According to data from Mobile Health Map, a database for mobile clinics in the United States, 24% of mobile clinics are affiliated with a university and 29% are affiliated with health care systems [10, 33]. The MCHC’s lack of formal affiliation with a health system meant referrals were dependent on providers’ familiarity with the geographic region, local networks of providers, and their sponsoring organization’s resources. This system places less connected or experienced providers at a disadvantage. Therefore, our findings suggest that having a formalized process and/or building partnerships with existing community healthcare centers has the potential to streamline referrals and allow for care continuation for marginalized communities regardless of a provider’s professional network. On the other hand, those who are likely to utilize the services of a mobile community health clinic may have developed a special relationship with independent MCHCs and value their independence from traditional large medical facilities. Therefore, retaining independence and autonomy might be critical for maintaining trust.

Moreover, additional improvements to a mobile community clinic’s referral system can be strengthened with a rigorous needs assessment and increased collaboration between community partners and medical centers [31]. Same day referrals, which were endorsed by our study participants, have been shown to facilitate timely linkage to care in other populations, such as transgender women newly diagnosed with HIV [32]. In the MCHC setting, a client-centered approach would allow for clients to familiarize themselves with providers in their communities prior to establishing care with them.

In addition to the potential value of the partnership between mobile community health centers and larger medical centers, having dedicated community resources available on hand in these mobile clinics can significantly improve the referral to care process for marginalized and minoritized communities [30, 34, 35]. The providers and clients in this study suggested an updated list of SGM affirming providers that would help providers refer clients and help clients navigate referrals. While this could be the foundation on which an MCHC could start ensuring an improved referral system, the participants in our study also expressed that having an opt-in follow-up scheme could motivate clients and have them feel as though they were being supported in linking to care while at the same time also respecting their privacy and autonomy. One possible way to have follow-up and support is through a patient navigator system. In a study conducted in Massachusetts, patient navigators at a mobile clinic were instrumental in helping patients make health care appointments [30]. Given the current volunteer nature of the clinical services offered through the MCHC, as well as the importance of maintaining client privacy, the MCHC and similar clinics could benefit from adopting a structured referral approach and hiring and training racial, ethnic, sexual, and gender diverse client navigators to support clients who opt for medical referrals and follow-up communications.

### Limitations

Our sample was limited to one MCHC in rural South Carolina. While our findings are consistent with previously published evaluations of mobile community clinics, we believe that further exploration with other MCHCs in more urbanized areas could yield different findings. Second, the providers in our study did not reflect the diversity of the clients, so their perspectives and priorities might not fully represent that of the clients they seek to serve. Finally, our sample was limited to individuals who could complete the study in English; thus, our findings do not fully reflect the experiences of people who do not have English language proficiency.

## Conclusion

Both the clients and providers who participated in this study detailed their experiences with the medical referral process at the MCHC, with client experiences reflecting how providers navigate the referral process. While the MCHC helps create an entry point to health care services for marginalized and underserved communities, this form of clinical care is limited in its ability to provide comprehensive services for clients. Therefore, additional resources are needed to formalize the referral process and to ensure adequate and equitable delivery of care for all clients—regardless of a provider’s professional network and affiliated organization. Furthermore, providers and client navigators aligned with the MCHC’s mission can also serve as a bridge between the MCHC and specialty care providers.

There are opportunities for successful referrals to care at the MCHC. Based on our findings and the present literature around mobile health clinics, we offer the following recommendations for improving the referral processes at mobile clinics: (1) create a formalized process for referring clients to care; (2) recruit providers from the community, preferably those closely aligned with the community, in order to build rapport with clients; (3) hire peer navigators that can assist clients with the referral process and follow up with clients once a referral is made, and (4) adequately fund mobile clinics, which can be the first point of healthcare contact for persons, so they can provide infrastructure to promote continuity of care.

## Data Availability

The datasets used and/or analyzed during the current study are available from the corresponding author on reasonable request.

## Abbreviations

COVID-19: Coronavirus disease 2019
SGM: Sexual and/or gender minorities
MCHC: SGM-centered mobile community clinic
CBPR: Community-based participatory research
STI: Sexually transmitted infection
HIV: Human immunodeficiency virus
US: United States of America
